# Boosting Antimicrobial Activity of Ciprofloxacin by Functionalization of Mesoporous Silica Nanoparticles

**DOI:** 10.3390/pharmaceutics13020218

**Published:** 2021-02-05

**Authors:** Blanca de Juan Mora, Luís Filipe, Andreia Forte, Miguel M. Santos, Celso Alves, Fernando Teodoro, Rui Pedrosa, Manuela Ribeiro Carrott, Luís C. Branco, Sandra Gago

**Affiliations:** 1 LAQV-REQUIMTE, Departamento de Química da Faculdade de Ciências e Tecnologia, Universidade NOVA de Lisboa, Campus da Caparica, 2829-516 Caparica, Portugal; b.mora@campus.fct.unl.pt (B.d.J.M.); lm.filipe@campus.fct.unl.pt (L.F.); andie.fct@gmail.com (A.F.); miguelmsantos@fct.unl.pt (M.M.S.); 2 MARE—Marine and Environmental Sciences Centre, Politécnico de Leiria, Avenida Porto de Pesca, 2520-630 Peniche, Portugal; celso.alves@ipleiria.pt (C.A.); fjtdgm@gmail.com (F.T.); rui.pedrosa@ipleiria.pt (R.P.); 3 Centro de Química de Évora, LAQV-REQUIMTE, Instituto de Investigação e Formação Avançada, Departamento de Química, Escola de Ciências e Tecnologia, Colégio Luís António Verney, Universidade de Évora, 7000-671 Évora, Portugal; manrc@uevora.pt

**Keywords:** ciprofloxacin, ionic liquids, mesoporous silica nanoparticles, cytotoxicity, antimicrobial activity

## Abstract

Mesoporous silica nanoparticles (MSNs) are very promising nanomaterials for treating bacterial infections when combined with pharmaceutical drugs. Herein, we report the preparation of two nanomaterials based on the immobilization of ciprofloxacin in mesoporous silica nanoparticles, either as the counter-ion of the choline derivative cation (MSN-[Ch][Cip]) or via anchoring on the surface of amino-group modified MSNs via an amide bond (MSN-Cip). Both nanomaterials were characterized by TEM, FTIR and solution ^1^H NMR spectroscopies, elemental analysis, XRD and N_2_ adsorption at 77 K in order to provide the desired structures. No cytotoxicity from the prepared mesoporous nanoparticles on 3T3 murine fibroblasts was observed. The antimicrobial activity of the nanomaterials was determined against Gram-positive (*Staphylococcus aureus* and *Bacillus subtilis*) and Gram-negative (*Klebsiella pneumoniae*) bacteria and the results were promising against *S. aureus*. In the case of *B. subtilis*, both nanomaterials exhibited higher antimicrobial activity than the precursor [Ch][Cip], and in the case of *K. pneumoniae* they exhibited higher activity than neutral ciprofloxacin.

## 1. Introduction

The treatment of infectious diseases caused by bacteria is frequently achievable by the use of antibiotics; however, these diseases are still one of the major causes of death worldwide. Such a failure in antibiotic treatment may be due to the rise of drug-resistant bacteria strains, as well as insufficient antibiotic activity or concentration at the site of infection. These problems are related to the reduced bioavailability, permeability and drug delivery of the drugs currently used. Recently, an interesting class of compounds that combines active pharmaceutical ingredients (APIs) with biocompatible counter-ions, such as organic salts and ionic liquids (OSILs)—API-OSILs—has been reported as a promising alternative to improve the original drug properties in terms of crystallinity, stability, solubility, permeability and delivery [1,2]. In our research group, we have been involved in the preparation and characterization of API-OSILs from β-lactam [3,4,5,6,7], fluoroquinolone [7,8,9], antibiotics, NSAIDs (ibuprofen [7,10], naproxen [7]) and bone antiresorptive agents (zoledronic [11] and alendronic [12] drugs). More specifically, our works on API-OSILs based on fluoroquinolone drugs, namely ciprofloxacin and norfloxacin, focused on their combination as organic cations with methanesulfonate (Mes), gluconate (Glu) and glycolate (Gly) as anions [8], or acting as organic anions with organic cations such as pyridinium, ammonium and *N*-methylimidazolium moieties [9]. These works showed that, depending on the cation or anion–drug combinations, it is possible to tune important physical, thermal and biological properties, leading to novel organic salts with enhanced properties in terms of bioavailability, membrane affinity, permeation, polymorphism elimination and also antimicrobial activity.

The use of nanostructured platforms based on mesoporous silica nanoparticles (MSNs) has also been investigated as highly stable and efficient systems for the incorporation of different drugs to increase their efficiency [13]. In fact, MSNs possess several advantageous features, such as high specific surface area and volume, tuneable surface charge/pore size, high loading capacity, good biocompatibility, high chemical stability, and the easy functionalization of inner pore and surface, which allows the possibility of creating a great variety of morphologies and surface functionalities [13,14]. MSNs carrying antibiotics have been reported in the literature in combination with levofloxacin [15,16,17,18,19], gentamicin [20,21], isoniazid [22,23], moxifloxacin [24,25,26], ampicillin [27], kanamycin [28], tetracycline [29,30,31] and ciprofloxacin [32,33,34,35], and the results indicated that these systems can boost the antimicrobial efficacy and safety profiles of currently available antibiotics. In more detail, the works based on the combination of MSNs and ciprofloxacin have focused on surface functionalization with sulfonate [32,36], silane groups [37], lipids [35], amino acids [34], polymers [38,39] and also non-pathogenic magnetotactic bacteria [33], in order to increase drug loading capacity and promote a controllable and efficient drug delivery. Finally, the effect of different pore sizes and volumes has also been investigated as crucial factors for ciprofloxacin loading and release [40,41].

**Figure 1:**
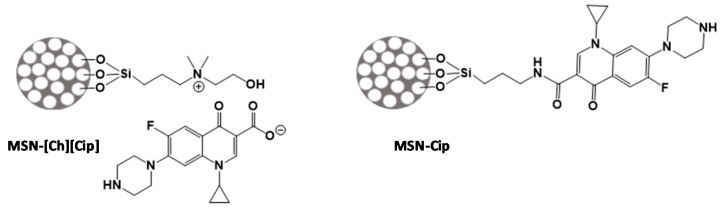
Ciprofloxacin immobilized in mesoporous silica nanoparticles (by functionalization of choline derivative cation, MSN-[Ch][Cip]) or by direct functionalization of ciprofloxacin drug MSN-Cip.

To the best of our knowledge, the incorporation of API-OSILs into MSNs remains unexplored in the literature. As such, in this work, ciprofloxacin (Cip) was immobilized in MSNs either as the counter-ion of a choline derivative cation (MSN-[Ch][Cip]), or was covalently anchored on the surface of an amino group-modified MSN via an amide bond (MSN-Cip), as shown in Figure 1. Our aim was to evaluate how these two different synthetic methods can affect the cytotoxicity and antimicrobial activity of the analogue API-OSIL ([Ch][Cip]) and Cip.

## 2. Experimental Section

### 2.1. Materials and Methods

Tetraethyl orthosilicate (TEOS, Aldrich, 98%), hexadecyltrimethylammonium bromide (CTAB, BDH Chemicals, Radnor, PA, USA), Pluronic F127 (Aldrich, Munich, Germany), triethanolamine (TEA, Alfa Aesar, Ward Hill, MA, USA), (3-chloropropyl)triethoxysilane (TCI, 97%), 2-dimethylaminoethanol (Aldrich, 99.5%) ciprofloxacin (Cipro, Fluka, 98%) and NaOH, 3-aminopropyl)triethoxysilane (APTES, Aldrich, 99%) were used.

1-(3-Dimethylaminopropyl)-3-ethylcarbodiimide HCl (EDC.HCl, Carbosynth) and *N*-hydroxysuccinimide (NHS, Aldrich, 98%) were purchased from chemical suppliers and used without further purification.

FT-IR spectra were recorded on a Bruker Tensor 27 Spectrometer in the 400–4000 cm^−1^ region, using KBr pellets. Solution ^1^H NMR spectra were obtained with a Bruker AMX400 at 400.13 MHz. Elemental analyses (EA) were carried out with a Thermofinnigan Flash EA 112 series. Transmission electron microscopy (TEM) images were acquired with a Hitachi H-8100 microscope with thermionic emission (LaB6) operated at 200 kV at MicroLAb (Instituto Superior Técnico, Lisboa, Portugal). The digital image acquisition was achieved from samples supported on carbon-coated copper grids with a CCD MegaView II bottom-mounted camera. Nanoparticles’ average diameters were achieved by manual analysis using ImageJ software (National Institute of Health, medical research agency, Bethesda, MD, USA). The X-ray diffraction (XRD) measurements were performed on a Bruker AXS-D8 Advance powder diffractometer, using CuKα radiation (40 kV, 30 mA), a step size of 0.01° (2θ) and 5 s per step. Nitrogen adsorption–desorption isotherms were determined at 77 K on a Quantachrome Quadrasorb equipped with a turbomolecular pump, using helium (for dead space calibration) and nitrogen of 99.999% purity. Before the adsorption measurements, the samples were outgassed at 120 °C for 8 h, using a heating rate of 1 °C min^−1^ to achieve the final temperature.

### 2.2. Description of the Synthesis

#### 2.2.1. Synthesis of Triethoxysilane Cholinium Derivative (Si-[Ch][Cl])

**Figure 2:**
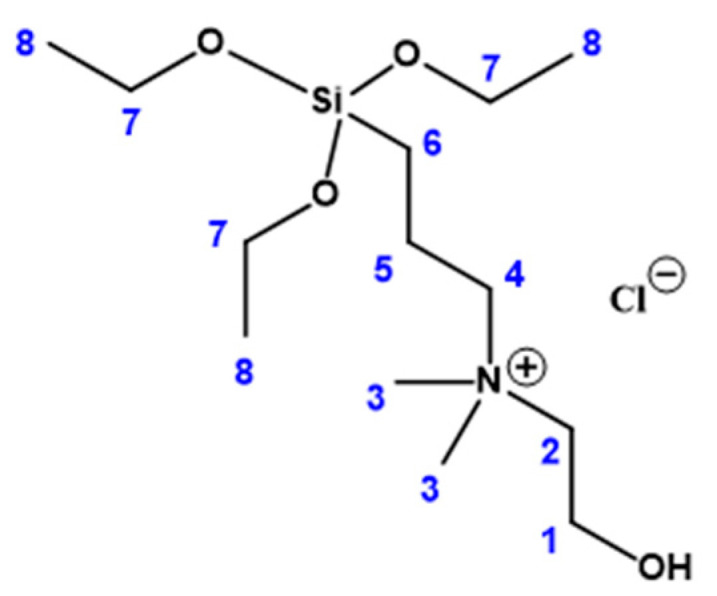
Numbering scheme for the assignment of the ^1^H NMR spectrum of Si-[Ch][Cl].

3-(chloropropyl)triethoxysilane (4.05 mL, 16.8 mmol) and 2-dimethylaminoethanol (1.69 mL, 16.8 mmol) were added to a round-bottomed flask. The reaction mixture was stirred for 24 h at 80 °C. The final mixture was then washed with diethyl ether and dried under vacuum to give the final product designated as Si-[Ch][Cl] (4.78 g, 14.5 mmol, 86%). ^1^H NMR (400.13 MHz, CDCl_3_, rt) δ = 4.13 (br, 2H, H-2), 3.84 (q, 6H, H-7), 3.71 (br, 2H, H-4), 3.53 (t, 2H, H-1), 3.37 (s, 6H, H-3), 1.87 (d, 2H, H-5), 1.24 (t, 9H, H-8), 0.65 (t, 2H, H-6) (see Figure 2 for numbering assignment). Selected FTIR (KBr/cm^−1^): 3379 (br); 3026 (w), 2950 (w), 2887 (w), 1633 (m), 1483 (s), 1085 (vs), 937 (w), 700 (m), 574 (m).

#### 2.2.2. Synthesis of Sodium Salt of Ciprofloxacin ([Na][Cip])

To a solution of ciprofloxacin (1 g, 3.02 mmol) in methanol (25 mL), a solution of sodium hydroxide (NaOH; 0.5 M, 6.04 mL) was slowly added. The solution was stirred for 24 h, the solvent was removed using a rotary evaporator and the final product was dried under a vacuum to obtain [Na][Cip] (1.07 g, 3.02 mmol).

#### 2.2.3. Synthesis of Mesoporous Silica Nanoparticles (MSNs) (MSN-OH)

The mesoporous nanosilica support was prepared as described by Bouchoucha et al. [42]. A mixture containing Pluronic F127 (2.68 g), CTAB (0.663 g), and TEA (15.64 g) in water (125 mL) and EtOH (57 mL) was prepared and stirred at room temperature for 24 h. Afterwards, TEOS (2.56 mL) was added for 1 min under vigorous stirring to achieve a global molar ratio of TEOS:CTAB:F127:H_2_O:EtOH:TEA of 1:0.16:0.017:605:84:9.16. The mixture was aged for 24 h under static conditions and then EtOH (100 mL) was added to promote precipitation. The resulting powders were obtained by centrifugation, washed with water and dried in the oven at 80 °C. The solid was calcined at 550 °C in air for 5 h at a heating rate of 1 °C/min. The selected FT-IR (cm^−1^) were 3448 (br), 1636 (s), 1087 (vs), 969 (w), 803 (s), 459 (s).

#### 2.2.4. Synthesis of Mesoporous Silica Nanoparticles Functionalized with Si-[Ch][Cl] (MSN-[Ch][Cl])

Calcined MSN (0.50 g) was heated at 150 °C under reduced pressure for 2 h to remove the physiosorbed water. After cooling down to room temperature, an excess of *N*-(3-triethoxysilylpropyl)cholinium chloride (Si-[Ch][Cl]) (0.4 g) in ethanol (20 mL) was added and the mixture was stirred under reflux (85 °C) and nitrogen atmosphere for 24 h. The reaction mixture was then centrifuged (5000 rpm, 15 min) and washed with ethanol four times. The resultant material was dried overnight at 80 °C. Anal. Found (%): C, 8.88; N, 1.02; H, 2.08; ^1^H-RMN (400.13 MHz, D2O + NaOH, rt) δ = 3.85 (t, 2H), 3.52 (q, 2H), 3.20 (m, 4H), 2.97 (s, 6H), 1.71 (br, 2H), 1.04 (t, 2H), 0.29 (t, 2H) ppm. Selected FTIR (KBr/cm^−1^): 3443 (br), 2964 (sh), 2922 (sh), 1638 (s), 1483 (m), 1384 (m), 1086 (vs), 963 (w), 802 (s), 460 (s).

#### 2.2.5. Synthesis of Mesoporous Silica Nanoparticles Functionalized with [Ch][Cip] (MSN-[Ch][Cip])

The material MSN-[Ch][Cl] (0.150 g) was suspended in water (10 mL) with stirring and the sodium salt of ciprofloxacin ([Na][Cip]) (0.150 g, 0.42 mmol) was added in 5 mL of water. The mixture was stirred at room temperature for 24 h, centrifuged (5000 rpm, 15 min) and washed with water four times. The resultant material was dried overnight at 80 °C. Anal. Found (%): C, 16.28; N, 2.70; H, 2.68; ^1^H-RMN (400.13 MHz, D_2_O + NaOH + DMSO, rt) δ = 8.43 (s, 1H), 7.85 (d, 1H), 7.55 (d, 1H), 3.87 (b, 2H), 3.56 (b, 2H), 3.18 (s), 2.99 (s), 2.93 (s), 1.73 (br, 2H), 1.28 (br), 1.04 (s), 0.31 (t, 2H) ppm. Selected FTIR (KBr/cm^−1^): 3460 (br), 2966 (sh), 2916 (sh), 2850 (sh), 1740 (w), 1628 (s), 1493 (m), 1383 (m), 1318 (w), 1089 (vs), 968 (w), 802 (s), 730 (w), 620 (w), 547 (w), 462 (vs).

#### 2.2.6. Synthesis of Mesoporous Silica Nanoparticles Functionalized with (3-Aminopropyl)triethoxysilane (APTES) (MSN-APTES)

Calcined MSN (0.50 g) was heated at 150 °C under reduced pressure for 2 h to remove the physiosorbed water. After cooling down to room temperature, toluene (15 mL) and an excess of (3-aminopropyl)triethoxysilane (0.5 mL) were added and the mixture was refluxed (120 °C) for 48 h. The reaction mixture was then centrifuged (5000 rpm, 15 min) and washed with toluene several times. The resultant material was dried overnight at 80 °C. Anal. Found (%): C, 8.52; N, 2.59; H, 2.17; ^1^H-RMN (400.13 MHz, D_2_O + NaOH, rt) δ = 2.43 (t, 2H), 1.36 (m, 2H), 0.32 (t, 2H). Selected FTIR (KBr/cm^−1^): 3443 (br), 2957 (w), 2848 (sh), 1636 (m), 1558 (w), 1472 (w), 1400 (w), 1385 (m), 1084 (vs), 962 (sh), 796 (m), 687 (w), 669 (w), 619 (w), 459 (s).

#### 2.2.7. Modification of MSN-APTES with Ciprofloxacin (MSN-Cip)

The conjugation of MSN-APTES with ciprofloxacin was done following the method described in the literature using EDC/NHS chemistry [43]. Briefly, ciprofloxacin (0.5 g, 1.51 mmol) was dispersed in 40 mL of a mixture of water and DMSO (1:1), and EDC (0.270 g, 1.41 mmol) and NHS (0.160 g, 1.40 mmol) were added. The mixture was stirred for 6 h and then a suspension of MSN-APTES (0.230 g) in 20 mL of 1:1 aqueous DMSO was added. The reaction mixture was stirred at room temperature for 48 h. The resultant solid was centrifuged (5000 rpm, 15 min) and washed with DMSO and ethanol several times. The resultant material was dried overnight at 80 °C. Anal. Found (%): C, 24.76; N, 4.99; H, 3.09. ^1^H-RMN (400.13 MHz, D_2_O + NaOH, rt) δ = 8.36 (s, 1H), 7.75 (s, 1H), 7.48 (s, 1H), 3.49 (br, 1H), 3.11 (s, 2H), 2.89 (s, 2H), 2.43 (t, 2H), 1.36 (m, 2H), 1.21 (d, 2H), 0.98 (br, 2H), 0.31 (t, 2H). Selected FTIR (KBr/cm^−1^): 3438 (br), 3082 (sh), 3042 (sh), 2958 (sh), 2928 (sh), 2856 (sh), 1625 (s), 1582 (w), 1550 (w), 1487 (m), 1379 (s), 1293 (w), 1089 (vs), 946 (m), 730 (w), 790 (m), 731 (m), 620 (w), 544 (w), 462 (s).

### 2.3. Cytotoxicity of Compounds

The cytotoxicity of the compounds synthesized was evaluated on 3T3 murine fibroblast cells previously acquired from the DSMZ biobank. Cells were cultivated according to the supplier instructions. Regarding the cytotoxicity assays, cells were seeded in 96-well plates at a density of 1.5 × 10^4^ cells/well and cultured for 3–5 days until reaching the total confluence. 3T3 cells were then treated with the compounds (100 µg/mL) for 24 h and the IC_50_ was determined (1–100 µg/mL; 24 h) for the most active (cell viability reduced >50%). The effects were estimated by the MTT (Sigma, Darmstadt, Germany) method and the results expressed in percentage of control (%).

### 2.4. Antimicrobial Activities

The antimicrobial activity of the compounds (100 µg/mL) was evaluated against *Bacillus subtilis* (ATCC 6633) and *Staphylococcus aureus* (ATCC 25923) grown in Lysogeny broth (LB) and *Klebsiella pneumoniae* (ATCC 11296) grown in Tryptic soy broth according to the protocol described by Santos and co-workers [9]. IC_50_ was determined (0.001–100 µg/mL) for the most active compounds.

### 2.5. Data and Statistical Analysis

The results are presented as mean ± standard error of the mean (SEM). Statistical analysis was performed using a one-way analysis of variance (ANOVA) with Dunnett’s multiple comparison of group means to determine significant differences relatively to the control treatment. Differences were considered significant at a level of 0.05 (*p*-value < 0.05). The determination of IC_50_ was performed by the analysis of non-linear regression by means of Equation (1):

(1) $$y=\frac{100}{\left[1+10\left(X-\log\left({\mathrm{IC}}_{50}\right)\right)\right]}$$

Calculations were performed using GraphPad v5.1 (GraphPad Software, La Jolla, CA, USA) software.

## 3. Results and Discussion

### 3.1. Synthesis and Characterization

**Scheme 1:**
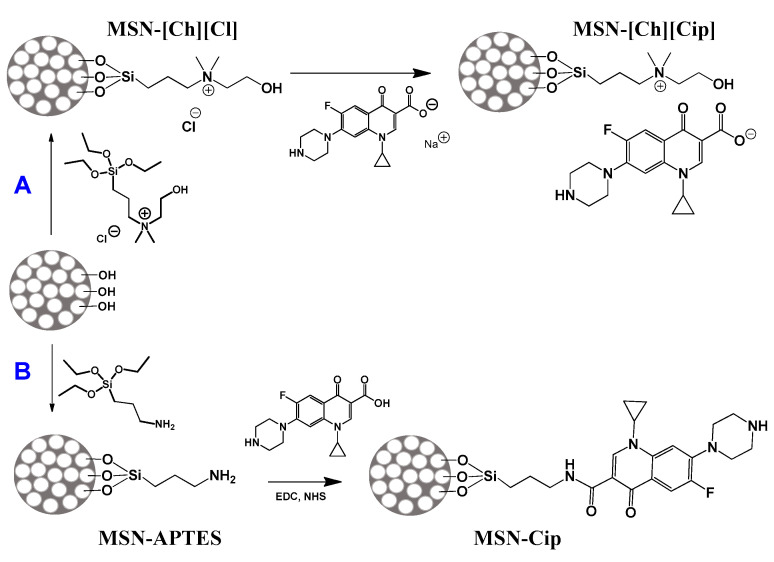
Synthetic approaches for the preparation of (**A**) MSNs with Cip as anion, MSN-[Ch][Cip] and (**B**) MSNs with Cip covalently anchored, MSN-Cip.

Mesoporous silica nanoparticles (MSNs) were prepared following the synthetic approach described by Bouchoucha et al. [42], using Pluronic F127 as the non-ionic surfactant and triethanolamine as the co-inhibitor of particle growth. These nanomaterials were functionalized to achieve ciprofloxacin (Cip)-loaded MSNs either in ionic or neutral form, following the two different synthetic routes A and B illustrated in Scheme 1.

For the immobilization of Cip as the anion (A), MSNs were covalently functionalized with a choline derivative cation (MSN-[Ch][Cl]), and then, by reaction with the sodium salt of Cip, the chloride was exchanged for anionic Cip (MSN-[Ch][Cip]). To obtain MSNs covalently modified with Cip in its neutral form (MSN-Cip), the nanoparticles were firstly modified with 3-aminopropyltriethoxysilane (MSN-APTES), and then using EDC/NHS (1-ethyl-3-(3-dimethylaminopropyl)carbodiimide/*N*-hydroxysuccinimide) coupling for the formation of amide bonds (by the condensation of the amine groups with the carboxylic groups of Cip).

The nanomaterials were characterized by TEM, FTIR and solution ^1^H NMR spectroscopies, elemental analysis, XRD and N_2_ adsorption–desorption experiments at 77 K.

**Figure 3:**
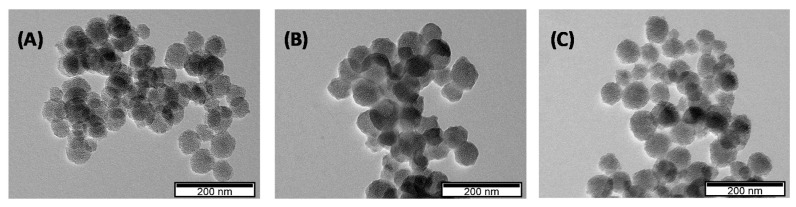
TEM images of (**A**) precursors MSN-OH, (**B**) MSN-Cip and (**C**) MSN-[Ch][Cip].

The TEM images show that MSNs are composed of spherical nanoparticles with an average diameter of 50 ± 11 nm, and that the functionalization procedures do not affect their morphology (Figure 3).

**Figure 4:**
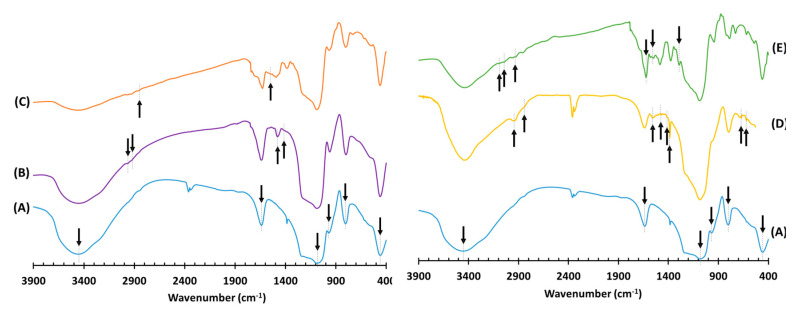
FTIR spectra of (**A**) pristine MSNs, (**B**) MSN-[Ch][Cl], (**C**) MSN-[Ch][Cip], (**D**) MSN-APTES and (**E**) MSN-Cip.

The FTIR spectra of the pristine and functionalized nanomaterials are exhibited in Figure 4. The MSN-OH spectrum shows the typical vibrations of the silica network at 1087, 969, 803 and 459 cm^−1^, which are attributed to ν_as_(Si–O–Si), ν_as_(Si–OH), ν_s_(Si–O–Si) and δ(Si–O–Si), respectively [44]. The peaks at 3448 and 1636 cm^−1^ are associated with the ν(O–H) and δ(O–H) of adsorbed water molecules, although the former can also be due to surface silanol groups. The spectrum of MSN-[Ch][Cl] shows bands at 2964 and 2922 cm^−1^, corresponding to ν(C–H), and at 1480 and 1384 cm^−1^, characteristic of the δ (C–H) of methyl groups of choline [45,46,47]. The band at 1638 cm^−1^ increases its intensity due to the overlap of δ(O–H) the vibrational modes from choline and adsorbed water. In the case of MSN-[Ch][Cip], besides the changes in the aromatic stretching region, the peak at 1520 cm^−1^ can be attributed to the asymmetric stretching vibration of COO^−^. The MSN-APTES spectrum contains additional bands at 2957 and 2848 cm^−1^ assigned to the ν(C–H) of the aliphatic chain, at 1558 and 669 cm^−1^ due to the δ(N–H) of the primary amine, and at 1472, 1400, 1385 and 619 cm^−1^ attributed to δ(C–H) [44,48]. The MSN-Cip spectrum exhibits new bands at 3082, 3042 and 2928 cm^−1^ assigned to the aromatic ν(C–H) of Cip moieties [49,50]. The peak at 1625 cm^−1^ is sharper and shifted when compared with the precursor material MSN-APTES, which could be due to the overlapping of the ν(C=O) of the pyridone ring of Cip with the δ(O–H) of the adsorbed water. The characteristic amide bands are found at 1550 and 1293 cm^−1^ for the N–H in-plane bend and C–N stretching, respectively [43].

The loading of anchored guests was estimated by elemental analyses. For MSN-[Ch][Cl], a content of 0.73 mmol g^−1^ of choline derivative cation was obtained (based on nitrogen analysis), and the C/N ratio is 10.2, which means that the reaction with the surface mainly occurs through two or one ethoxy groups. For MSN-[Ch][Cip], assuming 0.73 mmol g^−1^ as the initial content of [Ch][Cl] and using the nitrogen analysis that gives a total of 1.9 mmol g^−1^ of N, we can estimate that around 53% of Ch^+^ has Cip^−^ as the counter-ion, considering that *n*[Ch][Cl] + *n*[Ch][Cip] *×* 4 *=* 1.9 mmol, and that *n*[Ch][Cl] *=* 0.73 *× x* and *n*[Ch][Cip] *=* 0.73 *× y*, where *n* is the number of moles, *x* is the fraction of [Ch][Cl] that remains in the final material and *y* is the fraction of [Ch][Cip] and *x + y =* 1. In this hybrid, the C/N ratio is 7.2, which is close to the theoretical value 7.6, considering the guests molar fractions estimated and that the reaction with the surface occurred through two ethoxy groups. MSN-APTES reveals a loading of 1.85 mmol g^−1^ (based on nitrogen analysis), and the C/N obtained ratio is 3.83, which means that some of the anchored guests are bonded to the surfaces of MSNs through two ethoxy groups. In the case of MSN-Cip, elemental analysis indicates a total content of 3.6 mmol of N; if all APTES were converted to amide, we should have 7.4 mmol of N, and this suggests that only a fraction was converted. Assuming 1.85 mmol as the initial content of APTES and using the same approach explained above (in this case, *n_APTES_ + n_amide_ ×* 4 *=* 3.6 mmol, and that *n_APTES_ =* 1.85 *× x* and *n_amide_ =* 1.85 *× y*, and *x + y = 1*, where *x* is the fraction that remains in the material and *y* is the fraction that is converted into amine), we can estimate that around 32% was successfully converted to the desired amide. The C/N ratio obtained is 5.6, and the theoretical value is 5.2 if all the guests are anchored through two ethoxy groups, which indicates that the residual ethoxy groups in MSN-APTES increase with this last functionalization.

**Figure 5:**
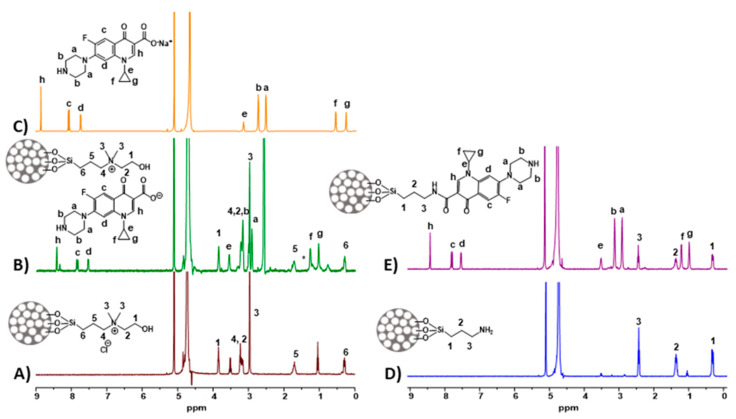
Solution ^1^H NMR of (**A**) MSN-[Ch][Cl], (**B**) MSN-[Ch][Cip], (**C**) Na[Cip], (**D**) MSN-APTES and (**E**) MSN-Cip in D_2_O + NaOH.

The functionalized nanomaterials were also characterized by solution ^1^H NMR spectroscopy, after dissolving the silica matrix, following the method described by Crucho et al. [51] (Figure 5). For MSN-[Ch][Cl] and MSN-APTES, loadings of 0.67 mmol g^−1^ and 1.66 mmol g^−1^ of anchored guests were obtained, respectively, by this method (using trioxane as internal reference), which are close to those obtained by elemental analyses. For MSN-[Ch][Cl], the peaks corresponding to choline derivative guests are identified in Figure 5A. In the case of MSN-[Ch][Cip], additional peaks in the aliphatic and aromatic regions are observed (Figure 5B), which are consistent with the anionic Cip ^1^H NMR spectrum (Figure 5C). In the case of MSN-Cip, several peaks corresponding to Cip moiety are identified, but the presence of the primary amine is also observed (denoted as *), which corroborates the partial conversion of the amine to amide.

**Figure 6:**
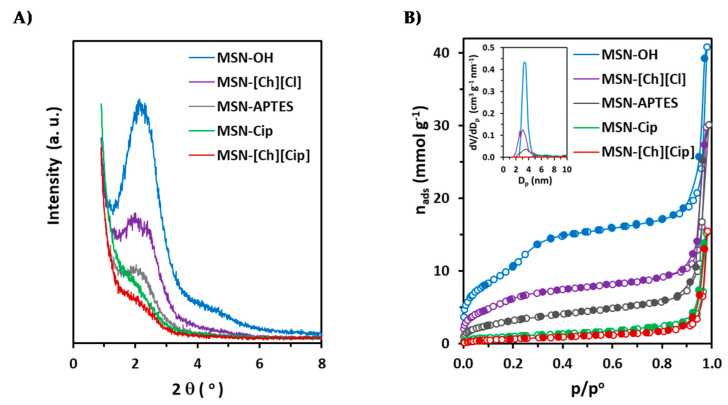
X-ray diffraction patterns (**A**) and nitrogen adsorption–desorption isotherms at 77 K (**B**) of MSN-OH, MSN-[Ch][Cl], MSN-[Ch][Cip], MSN-APTES and MSN-Cip. NLDFT pore size distributions are inserted into (**B**).

The pristine MSN-OH and the functionalized nanomaterials were also characterized by XRD and N_2_ adsorption–desorption isotherms at 77 K (Figure 6). As can be seen in Figure 6A, the X-ray diffraction pattern of the precursor nanomaterial MSN-OH has a main peak in the low-angle region at around 2° (2θ), and another with much lower intensity around 4° (2θ), indicating some ordering of the pores with reasonably uniform pore size. The main peak is broader, less intense, and corresponds to d-spacing larger than that usually obtained for MCM-41 and MCM-48 silicas with similar average pore diameters, which is consistent with the less ordered arrangement of the pores inside such small nanoparticles. Nevertheless, it is remarkable that structural ordering was obtained, and our results confirm that the method developed by Bouchoucha et al. [42] and used for the synthesis of MSN-OH is appropriate to obtain ordered mesopores inside such small nanoparticles. The intensity of the diffraction peaks is reduced in the materials MSN-[Ch][Cl] and MSN-APTES, and even more so in the final materials MSN-[Ch][Cip] and MSN-Cip, which confirms the successful functionalization of the MSNs mesopores. In addition, it is noted that the XRD pattern corresponding to MSN-[Ch][Cl] presents two peaks at around 2° (2θ), suggesting that the choline derivative cation is not evenly located inside the mesopores. This splitting of the main peak is not perceptible in the case of MSN-APTES, but the XRD broad peak is much less intense, which is consistent with the larger amount of APTES introduced. Regarding the final materials, it is interesting to note that the intensity decreases less in the case of MSN-Cip, despite the larger amount of Cip, which suggests that Cip is, at least partially, located at some pore entrances in this material.

The N_2_ adsorption–desorption isotherms determined at 77 K are presented in Figure 6B, and the results of the analysis via the BET method, using criteria recommended by Rouquerol et al. [52] and subsequently endorsed by IUPAC [53], and by NLDFT using the Quantachrome software ASiQwin, are summarized in Table 1.

As can be seen in Figure 6B, the isotherm obtained on MSN-OH is type IV of the IUPAC classification [53], with a well-defined step associated with the filling of cylindrical mesopores by capillary condensation. The pore size distribution, inserted into Figure 6B, shows that the pore diameter is reasonably uniform. The nitrogen adsorption–desorption isotherm on the silica nanoparticles is reversible up to high pressure, and above 0.9 shows a hysteresis cycle that is usually obtained on materials with small size spherical nanoparticles and is associated with capillary condensation in voids between spherical particles. The modifications did not affect the shape of the hysteresis cycle, which is similar for all materials. These results indicate that the particles are spherical in shape and that the morphology has not been altered by the functionalization procedures, which is in accordance with the observations by TEM.

Regarding the nitrogen adsorption and condensation in the uniform mesopores, a reduction in the adsorbed amounts was obtained for nanomaterials MSN-[Ch][Cl] and MSN-APTES, with the effect being less pronounced for the former, in accordance with the smaller amount of the choline derivative cation. In fact, the nitrogen adsorption isotherm on MSN-[Ch][Cl] still presents a pore-filling step, which is displaced to lower relative pressure due to the decrease in pore size as obtained from NLDFT. In the case of MSN-APTES, the slightly larger mean pore size results from the disappearance of pores of smaller sizes, as seen in the pore size distribution. The decrease in specific surface area and pore volume, and the changes in the pore size distributions, are consistent with the XRD results, and indicate that anchored guests are inside mesopores, and that the modification with APTES in larger amounts than the choline derivative cation provoked more alterations. Nevertheless, both modified nanomaterials still possess a capacity for loading the ciprofloxacin. The great decrease in specific surface area from the materials MSN-[Ch][Cl] and MSN-APTES to the final materials MSN-[Ch][Cip] and MSN-Cip, and the observation that the uniform mesopores became unavailable for nitrogen adsorption, confirm the functionalization with ciprofloxacin in ionic and neutral forms.

### 3.2. Cytotoxic and Antimicrobial Activities

**Figure 7:**
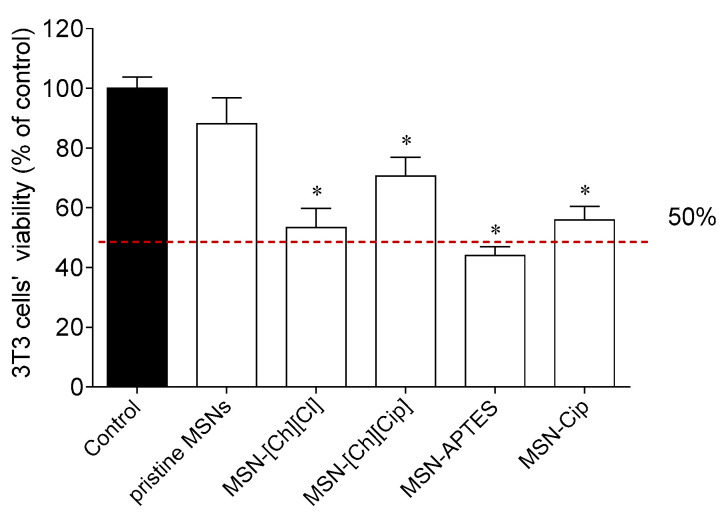
Cytotoxicity of compounds on 3T3 cells (100 µg/mL) after treatment for 24 h. Values represent mean ± standard error of the mean (SEM) of at least three independent experiments carried out in triplicate. Symbols represent significant differences (ANOVA, Dunnett’s test, *p* < 0.05) when compared to: * control.

The cytotoxicity of the prepared mesoporous nanoparticles on 3T3 murine fibroblasts was determined, and the results are exhibited in Figure 7. Among all materials tested, only the pristine MSNs compound did not reduce 3T3 cells’ viability at the maximum dose of 100 µg/mL. On one hand, this is in agreement with the biocompatible profile of MSNs. On the other hand, the anchoring of Cip, via ionic or covalent bonds, led to a reduction in cell viability of about 29% (71% of viable cells) and 41% (59% of viable cells), respectively. These data suggest that the IC_50_ values of both supported materials will be around, or higher than, 100 µg/mL, which is at least 581 times higher than the IC_50_ value observed for MSN-[Ch][Cip] against *K. pneumoniae*—the smallest IC_50_ value obtained for Cip-containing compounds (see below). Regarding Cip, in a previous study carried out by our research group, it did not affect 3T3 cells’ viability at concentrations below 10 μM [9]. Based on this, and once the highest antimicrobial activities were observed at concentrations substantially lower than 10 µM, its effects at concentrations above 10 μM were not tested.

Once MSN-APTES reduced 3T3 cells’ viability by more than 50% at 100 µg/mL, dose–response experiments were performed in order to accurately determine the corresponding IC_50_ value (Supplementary Materials Figure S1), which was found to be 9.63 µg/mL. However, there are also other studies reporting MSN-APTES nanoparticles without cytotoxicity at concentrations around 100 µg/mL [54]. This different result can be justified by the fact that similar materials may show distinct surface properties if they have different loadings of APTES, as it is known that particle structure, particle size and shape, functionalization and surface properties affect the cytotoxicity [55,56]. Although this first screening supplies relevant data about the cytotoxic effect of these prepared mesoporous nanoparticles on 3T3 cells’ viability, additional studies should be considered to depict the cytotoxic profile of the most promising compounds. For instance, the use of human cell lines, including primary cell cultures, as well as the establishment of more complex in vitro cellular systems, such as co-cultures and 3D cultures, should be considered to fully understand the potential cytotoxic effects of these compounds.

**Figure 8:**
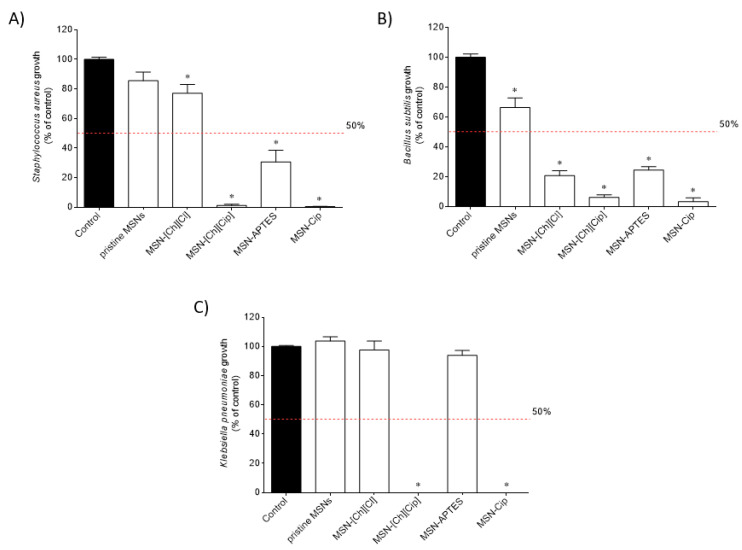
Growth inhibition of (**A**) *Staphylococcus aureus*, (**B**) *Bacillus subtilis* and (**C**) *Klebsiella pneumoniae* after exposure to the nanomaterials (100 µg/mL) for 7 h. Values represent mean ± standard error of the mean (SEM) of at least three independent experiments carried out in triplicate. Symbols represent significant differences (ANOVA, Dunnett’s test, *p* < 0.05) when compared to: *control.

The antimicrobial activity of the nanomaterials was determined against Gram-positive (*Staphylococcus aureus* and *Bacillus subtilis*) and Gram-negative (*Klebsiella pneumoniae*) bacteria, and the results are presented in Figure 8.

At 100 µg/mL, MSN-[Ch][Cip], MSN-APTES and MSN-Cip reduced the growth of *S. aureus* (Figure 8A) and *B. subtilis* (Figure 8B) by more than 50%. MSN-[Ch][Cl] also shows the ability to reduce *B. subtilis* growth by more than 50%. In the case of *K. pneumoniae* (Figure 8C), only the nanomaterials that contain ciprofloxacin, MSN-[Ch][Cip] and MSN-Cip, displayed this capacity. Therefore, dose–response assays were carried out for the most active samples. The results are presented in Figures S2–S4 and the corresponding IC_50_ values were determined (Table 2).

The smallest IC_50_ values against *Staphylococcus aureus*, *Bacillus subtilis* and *Klebsiella pneumoniae* were obtained for MSN-[Ch][Cip] and MSN-Cip, and these materials exhibited similar activity profiles. Gram-positive microorganisms (*S. aureus* and *B. subtilis*) were revealed to be the most sensitive to the materials tested. The most potent antimicrobial activities were observed at sub-toxic concentrations, according to the cytotoxicity studies performed on 3T3 cells. Taking into consideration the loading of MSN-[Ch][Cip] and MSN-Cip, a comparison between the antimicrobial activities of these materials and those previously obtained [9] for ciprofloxacin and the non-supported salt analogue [Ch][Cip] is feasible and is presented in Table 3. On one hand, it is possible to observe that the nanomaterials potentiate the antimicrobial activity against *S. aureus*. On the other hand, both nanomaterials exhibited a higher antimicrobial activity than the salt [Ch][Cip] against *B. subtilis*, which was, however, lower than the one found for free Cip. Finally, the referenced Cip-containing MSNs display higher activity than Cip against *K. pneumoniae*, yet lower than [Ch][Cip].

## 4. Conclusions

In conclusion, two nanomaterials were derived via the efficient immobilization of the ciprofloxacin in mesoporous silica nanoparticles. These nanomaterials were functionalized to achieve the desired ciprofloxacin (Cip)-loaded MSNs either in ionic or neutral form, following two optimized synthetic routes: (a) immobilization of ciprofloxacin in mesoporous silica nanoparticles, either as counter-ions of the choline-derivative cation (MSN-[Ch][Cip]), or (b) anchored on the surface of amino group-modified MSNs via an amide bond (MSN-Cip). For a complete characterization of both nanomaterials, different spectroscopic techniques such, as TEM, FTIR and solution ^1^H NMR spectroscopies, elemental analysis, XRD and N_2_ adsorption at 77 K were used.

After confirmation of the desired nanomaterial structures, biological studies were performed, including cytotoxicity and antimicrobial activity studies. The antimicrobial activity of the nanomaterials was determined against Gram-positive (*Staphylococcus aureus* and *Bacillus subtilis*) and Gram-negative (*Klebsiella pneumoniae*) bacteria, and the results are highly promising against *S. aureus*. In the case of *B. subtilis*, both nanomaterials exhibited higher antimicrobial activity than the precursor [Ch][Cip], and in the case of *K. pneumoniae*, they exhibit higher activity than neutral ciprofloxacin. These antimicrobial effects were observed at sub-toxic concentrations.

This suitable combination of mesoporous silica nanoparticles and neutral or ionic ciprofloxacin can be a promising therapeutic platform to treat different bacterial infections as novel crystalline formulations that may incorporate antibiotics, either in the free form or as ionic liquids and organic salts. In the latter case, such nanostructured materials may improve the physicochemical properties of these materials, namely their hygroscopic and amorphous profiles, as well as stability.

## Figures and Tables

**Table 1:** Textural characteristics of the nanomaterials obtained by analysis of the N_2_ adsorption isotherms determined at 77 K.

Sample	A_BET_ (m^2^ g^−1^)	D_p_ (nm)	V_p_ (cm^3^ g^−1^)
MSN-OH	821	3.30	0.46
MSN-[Ch][Cl]	485	3.06	0.21
MSN-[Ch][Cip]	56	-	0
MSN-APTES	257	3.54	0.09
MSN-Cip	80	-	0.01

**Table 2:** IC_50_ values (ng/mL) were determined for compounds (0.001–100 µg/mL) against the tested microorganisms and 3T3 cells. The values in parentheses represent the confidence intervals for 95%.

Nanomaterials	*S. aureus*	*B. subtilis*	*K. pneumoniae*	3T3
Pristine MSNs	>100,000	>100,000	>100,000	>100,000
MSN-[Ch][Cl]	>100,000	25,037 (19,856.0–31,571.0)	>100,000	>100,000
MSN-[Ch][Cip]	35.85 (31.95–40.21)	23.33 (19.79–27.51)	172.2 (128.6–230.6)	>100,000
MSN-APTES	>100,000	>100,000	>100,000	9630 (1360–68,150)
MSN-Cip	34.55 (23.98–49.78)	25.55 (21.74–30.04)	159.6 (121.8–209.1)	>100,000

**Table 3:** IC_50_ values (nM) for ciprofloxacin (Cip), non-supported [Ch][Cip] and for the nanomaterials containing Cip against the tested microorganisms. (To convert IC_50_ values of Table 1 into nM, we assumed estimated values of Cip moieties of 0.39 mmol g^−1^ in MSN-[Ch][Cip] and 0.59 mmol g^−1^ in MSN-Cip).

Compounds	*S. aureus*	*B. subtilis*	*K. pneumonia*
Cip [9]	29.16	3.84	196.5
[Ch][Cip] [9]	181.4	20.60	51.12
MSN-[Ch][Cip]	13.98	9.09	67.16
MSN-Cip	20.38	15.07	94.16

## Data Availability

Not Applicable.

## Supplementary Materials

The following are available online at https://www.mdpi.com/1999-4923/13/2/218/s1, Figure S1: Dose–response curves of compounds (1–100 µg/mL; 24 h) on 3T3 cells for IC_50_ determination. Values represent mean ± standard error of the mean (SEM) of at least three independent experiments carried out in triplicate. Figure S2: Dose–response curves of compounds (0.001–100 µg/mL; 7 h) against *Staphylococcus aureus* for IC_50_ determination. Values represent mean ± standard error of the mean (SEM) of at least three independent experiments carried out in triplicate. Figure S3: Dose–response curves of compounds (0.001–100 µg/mL; 7 h) against *Bacillus subtilis* for IC_50_ determination. Values represent mean ± standard error of the mean (SEM) of at least three independent experiments carried out in triplicate. Figure S4: Dose–response curves of compounds (0.001–100 µg/mL; 7 h) against *Klebsiella pneumoniae* for IC_50_ determination. Values represent mean ± standard error of the mean (SEM) of at least three independent experiments carried out in triplicate.
