# Supplementary material for: Boosting Antimicrobial Activity of Ciprofloxacin by Functionalization of Mesoporous Silica Nanoparticles
Source: Pharmaceutics. 2021 Feb 5;13(2):218. doi: 10.3390/pharmaceutics13020218 (PMC7914840; doi:10.3390/pharmaceutics13020218)
Supplement: Supplementary file 1 [file pharmaceutics-13-00218-s001.pdf]

pharmaceutics

MDPI

Supplementary Materials: Boosting Antimicrobial Activity of Ciprofloxacin by Functionalization of Mesoporous Silica Nanoparticles

Blanca de Juan Mora, Luís Filipe, Andreia Forte, Miguel M. Santos, Celso Alves, Fernando Teodoro, Rui Pedrosa, Manuela Ribeiro Carrott, Luís C. Branco and Sandra Gago

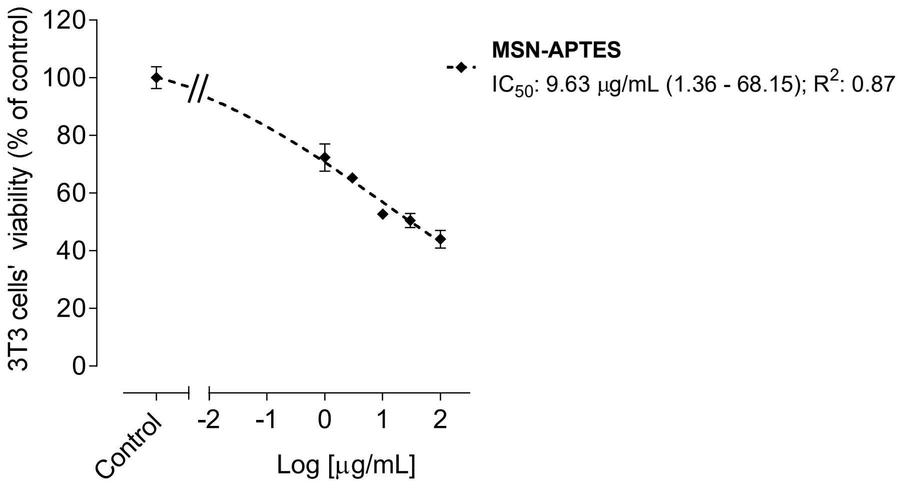

Figure S1. Dose-response curves of compounds (1–100 µg/mL; 24 h) on 3T3 cells for IC_{50} determination. Values represent mean ± standard error of the mean (SEM) of at least three independent experiments carried out in triplicate.

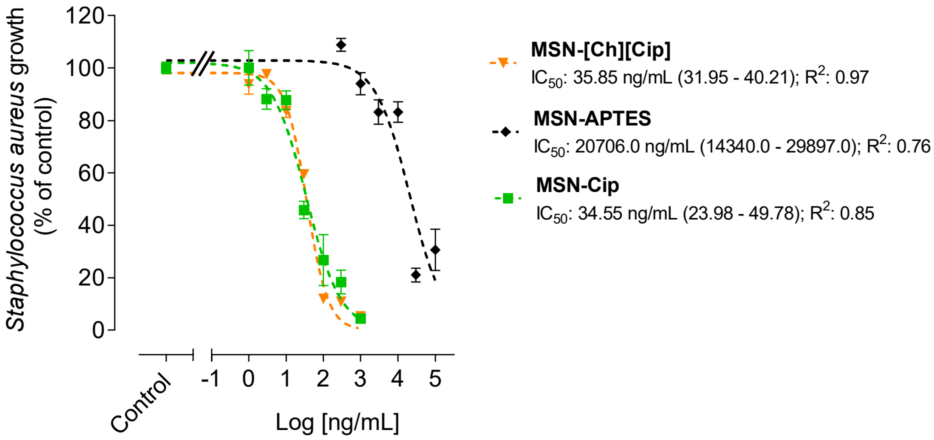

Figure S2. Dose-response curves of compounds (0.001–100 µg/mL; 7 h) against *Staphylococcus aureus* for IC_{50} determination. Values represent mean ± standard error of the mean (SEM) of at least three independent experiments carried out in triplicate.

Publisher's Note: MDPI stays neutral with regard to jurisdictional claims in published maps and institutional affiliations.

Copyright: © 2020 by the authors. Submitted for possible open access publication under the terms and conditions of the Creative Commons Attribution (CC BY) license (http://creativecommons.org/licenses/by/4.0/).

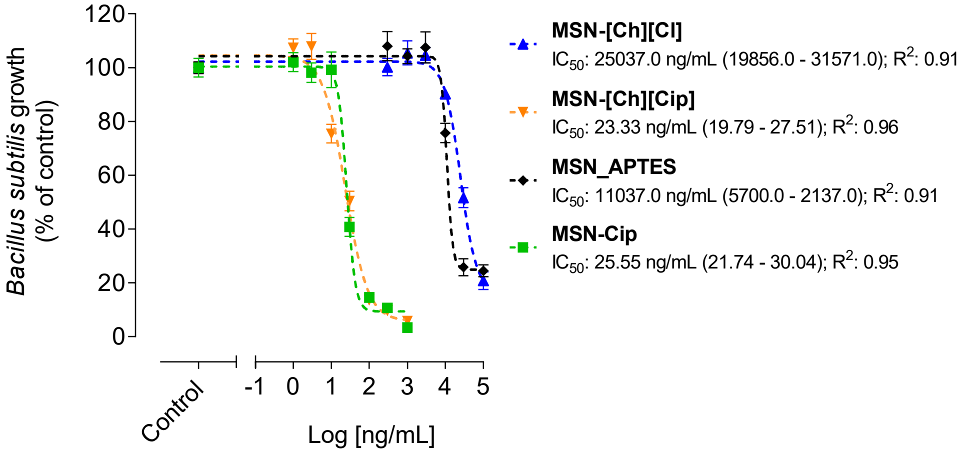

Figure S3. Dose-response curves of compounds (0.001–100 µg/mL; 7 h) against *Bacillus subtilis* for IC_{50} determination. Values represent mean ± standard error of the mean (SEM) of at least three independent experiments carried out in triplicate.

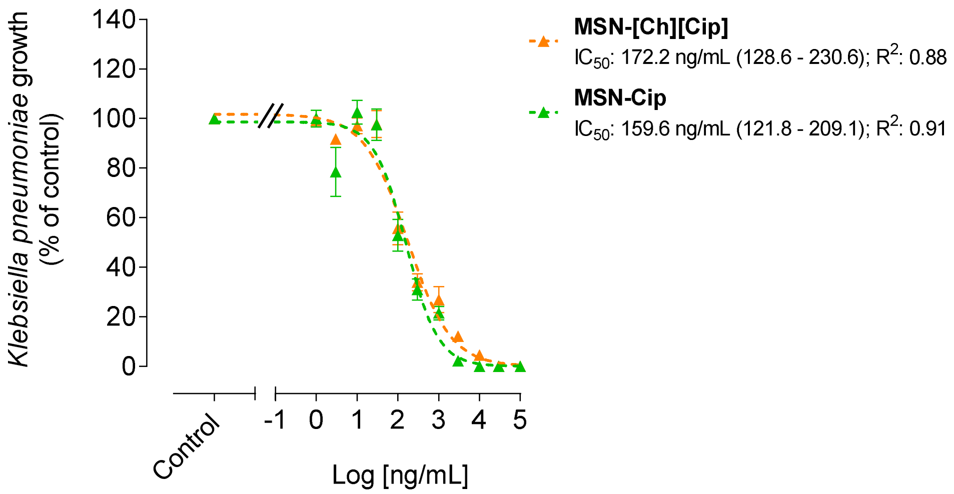

Figure S4. Dose-response curves of compounds (0.001–100 µg/mL; 7 h) against *Klebsiella pneumoniae* for IC_{50} determination. Values represent mean ± standard error of the mean (SEM) of at least three independent experiments carried out in triplicate.
